# 1082. Real-World Experience with Omadacycline for Nontuberculous Mycobacterial Infections: A Multicenter Evaluation

**DOI:** 10.1093/ofid/ofab466.1276

**Published:** 2021-12-04

**Authors:** Taylor Morrisette, Sara Alosaimy, Abdalhamid M Lagnf, Julie V Philley, Carly Sigler, Saira Butt, Emily A Kaip, Conan MacDougall, Carlos Mejia-Chew, Jeannette Bouchard, Jeremy J Frens, Tristan Gore, Yasir Hamad, Catessa Howard, Melissa Barger, M Gabriela Cabanilla, Aaron Ong, Michael P Veve, Andrew J Webb, Ryan W Stevens, Keira A Cohen, Michael J Rybak

**Affiliations:** 1 Anti-Infective Research Laboratory, Wayne State University, Detroit, Michigan; 2 Wayne State University, Detroit, Michigan; 3 The University of Texas Health Science Center, Tyler, Texas; 4 Indiana University School of Medicine, Indianapolis, Indiana; 5 University of California, San Francisco Medical Center, San Francisco, California; 6 University of California San Francisco School of Pharmacy, San Francisco, California; 7 Washington University in St Louis, St. Louis, Missouri; 8 University of South Carolina, Columbia, South Carolina; 9 Cone Health, Greensboro, NC; 10 Univeristy of South Carolina, Columbia, South Carolina; 11 Washington University, St. Louis, Missouri; 12 WVU Medicine, Morgantown, West Virginia; 13 Ventura County Medical Center, Ventura, California; 14 University of New Mexico Hospitals, Albuquerque, New Mexico; 15 Johns Hopkins University School of Medicine, Baltimore, Maryland; 16 Mayo Clinic, Rochester, Minnesota; 17 Wayne State University / Detroit Medical Center, Detroit, Michigan

## Abstract

**Background:**

Nontuberculous mycobacteria (NTM) are resistant to numerous antibiotics and lead to significant morbidity and mortality. Omadacycline (OMC) is an aminomethylcycline antibiotic that is Food and Drug Administration-approved for acute bacterial skin and skin structure infections and community-acquired bacterial pneumonia. Furthermore, OMC has shown *in vitro* activity against NTM. Given that real-world evidence is lacking, our primary objective was to evaluate the clinical success and tolerability of OMC when used for a variety of NTM infections.

**Methods:**

This was a multicenter, retrospective, observational study conducted from January 2020 to June 2021. We included all patients ≥ 18 years of age that received OMC of any indication for *Mycobacterium* spp. The primary outcome was clinical success, defined as a lack of all-cause mortality, lack of persistence or re-emergence of infection during or after therapy, and lack of alteration of OMC. Incidence of adverse effects potentially attributable to OMC and reasons for OMC utilization were also analyzed.

**Results:**

A total of 31 patients were included from 12 geographically distinct academic health systems (median age: 57 (IQR, 45-63) years; 45% male; 81% Caucasian). The majority of isolated pathogens were *Mycobacterium abscessus* complex (84%) and of those with subspeciation performed (54%), the majority (86%) were subsp. *abscessus*. The primary infections were of pulmonary origin (67%) and the median (IQR) duration of OMC therapy was 5.3 (3.2-9.4) months. Most isolates did not have OMC susceptibility conducted (87%), while the majority did for tigecycline (90%). Clinical success was reported in 81% of the population. Most patients were on combination antimicrobial therapy, and 39% of patients reported an adverse effect while on OMC (58% gastrointestinal distress). The majority of patients were prescribed OMC due to ease of administration (61%) and antimicrobial resistance to previous antibiotics (42%).

**Conclusion:**

OMC may be a potential option for the therapy of NTM infections. Prospective, randomized clinical trials are needed to confirm our preliminary findings.

**Disclosures:**

**Julie V. Philley, MD**, **Paratek Pharmaceuticals** (Advisor or Review Panel member)**Paratek Pharmaceuticals, Inc.** (Consultant) **Michael P. Veve, Pharm.D.**, **Cumberland** (Grant/Research Support)**Paratek Pharmaceuticals** (Research Grant or Support) **Michael J. Rybak, PharmD, MPH, PhD**, **Paratek Pharmaceuticals** (Research Grant or Support)

